# PLGA Nanoparticle-Based Formulations to Cross the Blood–Brain Barrier for Drug Delivery: From R&D to cGMP

**DOI:** 10.3390/pharmaceutics13040500

**Published:** 2021-04-06

**Authors:** Kaining Zhi, Babatunde Raji, Anantha R. Nookala, Mohammad Moshahid Khan, Xuyen H. Nguyen, Swarna Sakshi, Tayebeh Pourmotabbed, Murali M. Yallapu, Harry Kochat, Erene Tadrous, Shelby Pernell, Santosh Kumar

**Affiliations:** 1 Plough Center for Sterile Drug Delivery Solutions, University of Tennessee Health Science Center, 208 South Dudley Street, Memphis, TN 38163, USA; braji@uthsc.edu (B.R.); hkochat@uthsc.edu (H.K.); 2 Covance Inc., Kinsman Blvd, Madison, WI 53704, USA; anfh3@mail.umkc.edu; 3 Department of Neurology, College of Medicine, University of Tennessee Health Science Center, 855 Monroe Avenue, Memphis, TN 38163, USA; mkhan26@uthsc.edu; 4 Department of Pharmaceutical Sciences, University of Tennessee Health Science Center, 881 Madison Ave, Memphis, TN 38163, USA; xnguyen3@uthsc.edu (X.H.N.); ssakshi1@uthsc.edu (S.S.); etadrous@uthsc.edu (E.T.); pernellshelby@gmail.com (S.P.); 5 Department of Microbiology, Immunology and Biochemistry, College of Medicine, University of Tennessee Health Science Center, 858 Madison Avenue, Memphis, TN 38163, USA; tpourmot@uthsc.edu; 6 Department of Immunology and Microbiology, University of Texas Rio Grande Valley, McAllen, TX 78504, USA; murali.yallapu@utrgv.edu

**Keywords:** poly(lactic-*co*-glycolic acid) (PLGA), blood–brain barrier (BBB), current Good Manufacturing Practice (cGMP), Food and Drug Administration (FDA), nanotechnology

## Abstract

The blood–brain barrier (BBB) is a natural obstacle for drug delivery into the human brain, hindering treatment of central nervous system (CNS) disorders such as acute ischemic stroke, brain tumors, and human immunodeficiency virus (HIV)-1-associated neurocognitive disorders. Poly(lactic-*co*-glycolic acid) (PLGA) is a biocompatible polymer that is used in Food and Drug Administration (FDA)-approved pharmaceutical products and medical devices. PLGA nanoparticles (NPs) have been reported to improve drug penetration across the BBB both in vitro and in vivo. Poly(ethylene glycol) (PEG), poly(vinyl alcohol) (PVA), and poloxamer (Pluronic) are widely used as excipients to further improve the stability and effectiveness of PLGA formulations. Peptides and other linkers can be attached on the surface of PLGA to provide targeting delivery. With the newly published guidance from the FDA and the progress of current Good Manufacturing Practice (cGMP) technologies, manufacturing PLGA NP-based drug products can be achieved with higher efficiency, larger quantity, and better quality. The translation from bench to bed is feasible with proper research, concurrent development, quality control, and regulatory assurance.

## 1. Blood–Brain Barrier (BBB) and Drug Delivery

Compared with other therapeutic areas, drug development is more challenging for brain diseases such as brain cancers, Alzheimer’s diseases (AD), acute ischemic stroke, and human immunodeficiency virus (HIV)-1-associated neurocognitive disorders (HAND) [1,2,3,4]. Many systemically administered drug products cannot pass the BBB [5]. The BBB restricts the entry of compounds into the central nervous system (CNS) through the presence of brain microvascular endothelial cells, pericytes, perivascular astrocytes, and tight junctions. In addition, the presence of efflux transporters at the BBB has been recognized as a key element to poor drug penetration [6,7]. ATP-binding cassette (ABC) membrane-associated transporters, such as P-glycoprotein (P-gp), breast cancer resistance protein (BCRP), and multidrug resistance-associated protein (MRP1) show significant expressions at the BBB, protecting the brain from potential harmful endogenous and exogenous substances [6,7]. As a result, the BBB only selectively transports molecules such as certain amino acids, sugars, and gaseous molecules (e.g., oxygen and carbon dioxide) into the brain [8]. For example, antiretroviral drugs (ARVs) have shown to be effective in managing HIV-1 [9]. However, due to the inability of ARVs to cross the BBB, they are not highly recommended clinically for the treatment of HAND. Studies showed that, upon boosting with a pharmaco-enhancer, i.e., ritonavir, ARVs including indinavir, elvitegravir, and lopinavir reached therapeutic concentrations in plasma but did not reach therapeutic concentration in the brain, indicating the challenges of delivering drugs to the CNS [10,11].

## 2. Strategies to Cross BBB

Several strategies have been used to improve drug delivery to the brain. Efforts have been made for the development of inhibitors for ABC transporters due to their high expressions on the BBB [2,12,13]. Studies showed that blocking ABC transporters may significantly improve drug penetrations across the BBB. However, this method has not been used clinically due to the wide distribution of ABC transporters throughout the body, the potential toxicity of inhibitors, and unexpected drug–drug interactions [12]. Another approach is the “BBB opening” approach. Opening the BBB can be achieved by using a hyperosmotic solution to shrink the endothelial cells or using certain cytotoxic agents to disrupt the BBB tight junctions [12,13]. However, opening the tight junctions of the BBB is risky clinically because it may also allow the entry of harmful components into the brain and cause side-effects such as seizures and other long-term neurological complications [12]. Moreover, the development of prodrugs to increase their capacity to penetrate the BBB is another potential delivery approach [12]. Prodrugs can be synthesized with sufficient lipophilicity to facilitate the crossing of the endothelial cell membrane and release the parent ARVs into the brain. However, developing prodrugs as a delivery strategy needs a full evaluation of toxicity, cost, and efficacy, as prodrugs are considered to be a separate chemical entity.

A nanoparticle (NP)-based drug delivery system is considered a promising option to improve drug delivery to the brain [3]. NP-based formulations are usually a colloidal system made of polymers, lipids, or other large macromolecules such as albumin. A therapeutic agent may be released through diffusion or erosion of the matrix [14]. The NP-based delivery system can cross the BBB through membrane transcytosis, bypass efflux transporters, and effectively deliver the therapeutic molecule to the CNS [3]. NPs that have been studied for brain delivery include polymeric NPs such as poly(d,l-lactide-co-glycolide) (PLGA) [15,16] and poly(butyl-cyanoacrylate) (PBCA) NPs [17,18], magnetic NPs (MNPs) composed of an iron oxide core [18], lipid-based nanoformulations such as solid lipid nanoparticles (SLN) and liposomes [12,18], and polymeric micelles-based nanoformulations such as Pluronic micelles [12]. Extracellular vesicles (EVs), liposome-like natural carriers, have drawn attention for delivering drugs into the brain as a potential alternative to NPs [19,20,21].

## 3. Introduction of Physical, Chemical, and Biological Characteristics of PLGA Polymer

### 3.1. Synthesis of PLGA Polymer

PLGA is a synthetic copolymer composed of lactic and glycolic acid polyesters. Synthesis of PLGA is commonly achieved either through ring-opening polymerization reactions of lactide and glycolide [22,23] or through polycondensation reactions of lactic acid and glycolic acid to form PLA and PGA block polymers [24,25]. Ring-opening polymerization processes can be used to generate high-molecular-weight PLGA polymers [26], while polycondensation processes are more suitable for the synthesis of low-molecular-weight polymers [27].

### 3.2. Physicochemical and Biomolecular Characteristics of PLGA

PLA can exist in d- or l-lactic acid, as well as in d,l-lactic acid, configurations. Homo isomeric PLAs are more crystalline due to the uniform spatial arrangement leading to tighter packing of the polymer chains [28,29]. On the other hand, glycolic acid has no asymmetric carbon; thus, PGA exists only in a highly crystalline form. PLA is more hydrophobic than PGA due to its methyl side groups. As a result, the hydrophobicity and crystallinity of PLGA can be controlled through the ratio of lactide to glycolide. PLGA physicochemical properties, such as mechanical strength, solubility, rate of hydration, rate of hydrolysis, and glass transition temperature (Tg), are heavily influenced by crystallinity and hydrophobicity [30,31,32]. PLGA with a high degree of crystallinity will have a higher Tg and mechanical strength, as well as a decreased rate of hydration and hydrolysis.

In general, PLGA NPs are susceptible to clearance by the reticuloendothelial system (RES) through opsonin-mediated phagocytosis [33]. RES elimination and biodistribution of PLGA NPs depend on size, hydrophobicity, and surface charge. Cytotoxicity of PLGA NPs was investigated in vitro by monitoring the cell viability of Caco-2 and HeLa cell lines [34,35]. The study indicates that PLGA NPs at the concentration tested were not toxic to cells as both cell lines retained over 75% viability. According to histopathology assays, orally administered PLGA nanoparticles did not elicit adverse effects in mice [35]. Tissue distribution analysis in mice indicated that most of the PLGA NPs were detected in the liver, kidney, heart, and brain, with small amounts detected in plasma [35]. In aqueous conditions, PLGA undergoes hydrolysis of its ester bonds. Hydrolytic biodegradation of PLGA leads to nontoxic byproducts [36,37]. Several studies have investigated the factors that affect PLGA biodegradation, including intrinsic properties such as hydration rate, hydrophobicity/hydrophilicity, polymer chemical composition, molecular weight, crystallinity, and Tg [38]. Moreover, external factors such as pH and chemical additives were also shown to influence hydrolytic degradation [39].

## 4. PLGA NPs as a Brain Drug Delivery System

PLGA is a highly investigated polymer due to its ability to form NPs, micelles, and microspheres, as it possesses the properties of biocompatibility, biodegradability, and tolerability [40]. As drug delivery systems, PLGA NPs can be used to prepare controlled-release dosage forms of small-molecule drugs, peptides, and nucleic acids [41]. Through proper copolymerization with PEG and surface modifications with linkers, PLGA NPs have been demonstrated as promising carriers for drug delivery across the BBB.

### 4.1. PLGA NPs Modifications and Mechanisms

PLGA NPs can be prepared through various processing methods including (1) double-emulsion solvent evaporation [42], (2) single-emulsion solvent evaporation [43], (3) phase separation [44], (4) spray-drying [45,46,47], (5) salting out [48,49], and (6) nanoprecipitation [50,51]. Even though the names are different, processing methods focus on the self-assembly characteristics of PLGA in aqueous solutions to finish the drug encapsulation [52,53,54]. Details of processing methods and potential scale-up technologies are discussed in Section 5.

**Figure 1:**
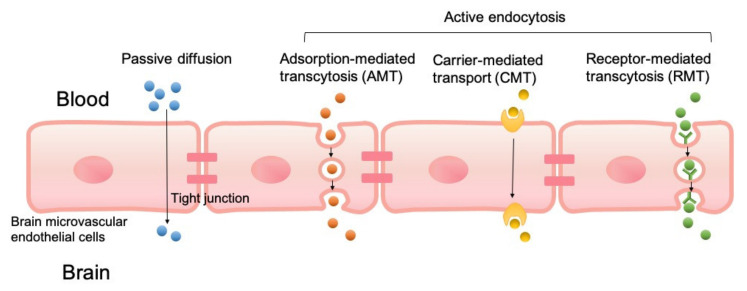
Transport mechanisms of poly(lactic-*co*-glycolic acid) (PLGA) nanoparticles (NPs) to cross the blood–brain barrier (BBB), including passive diffusion, adsorption-mediated transcytosis (AMT), carrier-mediated transport (CMT), and receptor-mediated transcytosis (RMT).

PLGA NPs can cross the BBB passively or through active endocytosis mechanisms as shown in Figure 1. Unmodified PLGA NPs cross the BBB primarily through passive internalization based on size, which was found to have low brain uptake. Several strategies have been developed to improve the penetration of NPs into the brain. These strategies modify NPs with components designed to take advantage of BBB endocytosis pathways. Modified PLGA NPs have been designed to cross the BBB through adsorption-mediated transcytosis (AMT) [55], carrier-mediated transport (CMT), and receptor-mediated transcytosis (RMT) [56].

PLGA NP surfaces are modified with positive charges that electrostatically interact with negatively charged regions of the luminal surfaces, which helps PLGA to cross the BBB. Several cationic modifications of PLGA NPs have been demonstrated to utilize the AMT concepts to improve brain uptake. In CMT systems, PLGA NPs are modified with membrane-permeable molecules such as amino acids, nutrients, and membranotropic peptides, and they are able to transport cargo across the BBB endothelium. CMT systems also include designs that take advantage of ABC transporters. With RMT, PLGA NPs are modified or covalently connected with ligands that target specific cell surface receptors known to be BBB transport pathways. A search of the PubMed database with the keywords “PLGA nanoparticles”, “BBB”, and “drug delivery” from 2015 to date returned 133 publications. From the search results, only research articles that included BBB permeability studies of control “unmodified” PLGA NPs and modified PLGA NPs are summarized in Table 1. Tandem systems that utilized multiple modifications have been reported. Guarnieri et al. [57] demonstrated the cooperative effects of glycoprotein H 625, a CMT modification with iron-mimicking protein CRT and RMT modification, in enhancing PLGA NP permeation of BBB. Liu et al. [58] developed a PLGA NP drug delivery system modified with angiopep-2 (RMT) and 1, 2-Dioleoyl-3-trimethylammonium-propane (AMT), for gefitinib and Golgi phosphoprotein 3 for the treatment of glioblastoma. Intranasal [59,60,61] or subcutaneous [62] administration of PLGA NP drug systems can bypass the BBB and avoid issues associated with systemic administration.

### 4.2. PLGA–PEG Co-Polymeric NPs, Modifications, and Applications

To overcome its short half-life, PLGA is combined with polyethylene glycol (PEG) to form PLGA–PEG copolymer NPs [63,64,65]. The PLGA–PEG copolymer is widely used in pharmaceutical products and devices. Recently, several advancements have been made to modify the surface of the PLGA–PEG NPs to further increase their ability to cross the BBB and deliver drugs into the brain.

The favorable chemistry of the PLGA–PEG NPs makes them amenable for conjugation with various peptides and linkers for use in the treatment of various neurodegenerative diseases and glioma. Memantine is commonly used for the treatment of mild and moderate AD. Encapsulating it in PLGA–PEG NPs using a double-emulsion method increased the delivery to the target tissue with ameliorated pathological markers compared to free memantine [66].

Pioglitazone-loaded PLGA–PEG NPs produced by the solvent displacement technique reduced amyloid burden and decreased memory impairment by increasing the rate of transcytosis across BBB and slowly releasing pioglitazone in the target tissue [78]. Selegiline- or donepezil-loaded PLGA–PEG NPs produced by the solvent evaporation method destabilized the beta-amyloid formation in vitro [79,80]. Various natural compounds and drugs encapsulated in PLGA–PEG NPs were shown to be effective in reducing AD pathology in in vitro models [81]. The antinociceptive effect of loperamide was increased by two- to threefold by encapsulation in a PLGA–PEG–PLGA triblock polymeric NPs coated with poloxamer 188 or polysorbate 80 compared to unmodified NPs alone [82]. With peptides as a modification on PLGA–PEG NPs, Hoyos-Ceballos et al. [35] showed that angiopep-2 conjugated to PLGA–PEG NPs increased their ability to cross the BBB in C57/BL6 mice. PLGA–PEG NPs conjugated with B6 peptide increased the delivery of curcumin into the CNS in an AD mouse model, showing a reduced expression of hallmark AD pathological markers, including amyloid-beta, presenilin-1, phosphorylated tau, and beta-secretase 1, compared to curcumin alone or NPs without B6 peptide [83].

Surnar et al. [84] added a targeting function to PLGA–PEG NPs through conjugating with a lipophilic triphenylphosphonium cation on the surface using a butylene linker. This NP system, when loaded with either coenzyme Q10 or aspirin, was able to cross the mitochondrial double membrane in endothelial cells and astrocytes to reduce the oxidative stress, which is extremely valuable to treat HAND [84]. Yu et al. [85] optimized the development of PLGA–PEG polymersomes conjugated on the surface with lactoferrin as a brain targeted delivery system for peptides. They loaded the NPs with S14G-humanin peptides which exerted a protective effect by decreasing the caspase-3 and bax expression in the rat hippocampus neurons treated with amyloid-beta. Similarly, Bi et al. [72] showed that PLGA–PEG NPs modified with lactoferrin on their surface were able to deliver rotigotine into the striatum by intranasal administration for potential use in Parkinson’s disease (PD). Lectin-conjugated PLGA–PEG NPs were able to deliver the basic fibroblast growth factor peptide cargo across the BBB after intranasal administration [86]. Similarly, odrranalectin-conjugated PLGA–PEG NPs were able to efficiently deliver the encapsulated urocortin peptide into the brain as a treatment for PD [86]. Recently, Amanda et al. [87] showed that a modified PLGA–PEG NP system encapsulating epigallocatechin gallate was able to ameliorate neurological deficits induced by 3-nitropropionic acid in Huntington’s disease mouse model.

Glioma is an invasive carcinoma of the brain with an average life expectancy of approximately 12–14 months and has poor survival rates [88]. NP systems were used to deliver drug into the brain. Receptor-mediated transcytosis provides a chance to target specific receptors expressed on the surface of cancer cells through ligand–receptor interactions. Cui and coworkers [89] developed a novel dual-targeting PLGA–PEG-based magnetic NP system that crosses the BBB by conjugating transferrin receptor-binding peptide T7 on the surface and encapsulating curcumin and paclitaxel into the NP hydrophobic core. Compared to free drugs, the mice with orthotopic glioma survived with the NP system. Similarly, doxorubicin and tetrahydrocurcumin encapsulated in transferrin-modified PLGA–PEG NPs showed effectiveness in reducing the glioma tumor volume in combination with radiotherapy [90]. Lactoferrin-conjugated PLGA–PEG NPs increased the brain concentrations of shikonin, a naphthoquinone pigment for the potential use in the treatment of glioma [91]. The iNGR-conjugated PLGA–PEG-based NP system designed to target the glioma tumor vessel was effective in delivering the paclitaxel to the glioma parenchyma. Moreover, this NP system was able to travel deeper into the glioma to increase survival rates [92]. Farnesyl thiosalicylic acid, an inhibitor of Ras oncoprotein, was shown to be effective against glioblastoma when administered as PLGA–PEG-based hybrid NPs, which contained 1,2-distearoyl-glycerol-3-phosphoethanolamine and 1,2-dioleoyl-3-trimethylammonium-propane [93].

Another strategy to treat glioma is to target the genes overexpressed in malignant glioma cells. Cyclic hexapeptide-conjugated PLGA–PEG NPs were able to deliver curcumin to the glioma by binding to integrins that are upregulated on the glial cell surface [94]. A nine amino acid linear peptide (Pep-1) targeting the interleukin 13 receptor α2 overexpressed on the surface of gliomas was conjugated to PLGA–PEG NPs [95]. Modification with CGKRK peptide, which targets the heparan sulfate expressed on neovascular endothelial cells, resulted in a dual-targeted approach and increased the median survival time in intracranial glioma mice [96]. A biodegradable PLGA–PEG polymer NP system targeting the Fn14 receptor overexpressed on the brain tumor cells was generated by conjugating PLGA–PEG NPs with ITEM4 monoclonal antibody. The half-life of these NPs was more than doubled compared to nontargeted PLGA–PEG NPs [97].

### 4.3. PLGA NPs for Theranostic Applications

Theranostic represents a novel and powerful emerging platform that integrates targeted therapeutic entities with noninvasive imaging and has the great potential to personalize and advance medicine. Several nanosized delivery vehicles, including gold and iron-oxide nanoparticles, as well as quantum dots, have been extensively studied for theranostic applications [98,99,100]. Given safety concerns, off-target effects, and slow excretion kinetics from the body, risks may limit its use in the course of diseases. PLGA NP-based theranostic applications can deliver a therapeutic agent while simultaneously monitoring therapy response in real time [101,102]. Contrast agents, such as the radionuclide or fluorophore, play a critical role in enabling visualization of a target with conventional imaging techniques, e.g., magnetic resonance imaging (MRI), optical imaging, and X-ray computed tomography. Contrast agents including superparamagnetic iron oxide (SPIO) and gadolinium were shown to be encapsulated with polymeric nanoparticles [103]. For example, SPIO and chemotherapy drug docetaxel can both be directly encapsulated with PLGA [104]. Similarly, an human epidermal growth factor receptor 2 (HER2)-targeted PLGA–PEG block copolymer nanoparticle, upon encapsulation with MnFe_2_O_4_ and doxorubicin, was designed to target breast cancer in vivo [105]. Encapsulation of anticancer drug N′-(2-Methoxybenzylidene)-3-methyl-1-phenyl-H-Thieno[2,3-c]Pyrazole-5-Carbohyd-razide (MTPC) with plasmonic gold nanorods in PLGA-*b*-PEG polymeric nanospheres enhanced both biodistribution and pharmacokinetics of the MTPC in tumor-bearing mice [106]. Similar to MRI, radionuclide imaging has high sensitivity with no tissue-penetration limitations. Several radionuclide compounds have been extensively studied along with PLGA with the goal of formulating a robust nano delivery system [107]. For instance, Wang and colleagues [108] designed PLGA-lipid hybrid nanocarriers for theranostic therapy, encapsulating an anticancer agent in the matrix, while the lipid shell was chelated with indium-111 or yttrium-90 as radiotherapy agents. Shao et al. [107] demonstrated the therapeutic effect of 32P-CP-PLGA brachytherapy for glioma with the integrin αvβ3-targeted radiotracer 68Ga-3PRGD2. Press and colleagues [109] demonstrated the cell-type-specific delivery of short interfering RNAs by covalent conjugation of DY-635 fluorescent dye with known hepatobiliary clearance to a PLGA, which allowed them to monitor distribution, uptake, and clearance of short hairpin RNA from the target organ. The major hurdle for the treatment of neurodegenerative diseases is to design therapeutic molecules in a way that it can cross the BBB. Zhang et al. [86] showed that the lectin-modified PLGA nanoparticle encapsulated with basic fibroblast growth factor enhanced drug delivery to the brain, supporting the role of the PLGA NP-based drug delivery system for CNS disorders. Therefore, nanocarriers based on PLGA offer biocompatibility, good stability, and regulated drug release rate and represent an excellent and emerging platform in theranostic medicine.

## 5. From Research and Development (R&D) to cGMP: Technologies for Scale-Up

To promote promising formulations from the R&D stage to clinical trials, drug products need to be manufactured under certain guidelines. Current Good Manufacturing Practice (cGMP) are regulations enforced by the FDA to ensure the quality of pharmaceutical products [110]. Most PLGA NP-based pharmaceutical products or medical devices are either injectable or implantable [111]. Therefore, sterility and potency are among the top-quality aspects to be considered for PLGA NPs. Per cGMP regulations, sterile products must be manufactured in a registered sterile facility [19]. Furthermore, facilities need to show that a sterile environment is properly maintained with validated sanitization [112]. PLGA NP-based pharmaceutical products have several steps during production: (1) dissolution and mixture of the active pharmaceutical ingredient (API) and PLGA; (2) stabilization of the mixture and removal of organic solvents; (3) separation of free API from PLGA–API product; (4) sterilization; (5) fill-finish. Table 2 presents selected publications showing processing details in the R&D stage.

For scale-up, dissolution of API/PLGA is achieved using stainless-steel tanks with temperature control through double jacketing. Mechanical motors are built into most tanks for light agitation. Since PLGA is not water-soluble, while lipophilic APIs can be dissolved concurrently, hydrophilic ones need to be dissolved separately from PLGA. To stabilize the formulation, PVA is mostly used as a surfactant. Moreover, poloxamer (also named Pluronic), polysorbate, sodium cholate, and D-α-tocopheryl pol-yethylene glycol succinate (TPGS) are also reported as promising alternatives. Subsequently, aggressive agitation is needed to decrease the mixture droplets’ particle size. High-pressure homogenization is widely used in cGMP production, and this technology is also reported with details in Table 2. To remove organic solvents, most publications took advantage of the different boiling points in water and organic solvents through overnight stirring. While this method is useful on the R&D scale, GMP-scale production uses vacuum-assisted rotary evaporation, which is more efficient and easier to validate.

Since PLGA NP-based pharmaceutical products represent a self-assembly drug delivery system, it is critical to separate free APIs from encapsulated ones. The encapsulation rate is one key quality control aspect due to the concern of toxicity. In Table 2, most publications used centrifugation, assisted by filters and membranes. In cGMP production, diafiltration is the technology preferred due to its higher recovery rate and better cost-effectiveness compared to centrifugation. Diafiltration uses a semipermeable membrane to separate the free drug from encapsulated drug based on the difference in cutoff molecular weight. Furthermore, diafiltration can form a closed system to return encapsulated products to the container for reprocessing until a target encapsulation rate is reached with the highest yield [113].

Among popular terminal sterilization methods, steam sterilization (autoclave) and gamma irradiation were reported to cause degradation of PLGA [114]. Vaporized ethylene oxide is commonly used for sterile gowning materials, but its residual is toxic if injected [115]. Electron beam technology is less aggressive than gamma irradiation but can still cause degradation of PLGA [116]. According to the available technologies for cGMP production and the particle size distribution reported from literature, sterile filtration is the best option for PLGA NP-based products. However, a filter compatibility study needs to be performed for validation purposes to minimize API retaining during filtration.

Depending on the concentration of PLGA NP-based pharmaceutical products, the solution may be free-flowing or with high viscosity. High viscosity is a problem for both sterile filtration and automatic filling systems. Filters may be clotted during filtration, the filling accuracy may be compromised, and rejection of vials may occur frequently. Therefore, an engineering fill-finish of placebo is strongly recommended as an industry standard for PLGA NP-based drug products. According to the stability data, lyophilization is recommended for extended shelf life. As presented in Table 2, lyophilization was reported using sugar molecules as cryoprotection. In cGMP production, lyophilization is considered a sterile product and is performed in the last step. The difficult part of lyophilization is the programming of cycles to minimize the moisture content (<5%, *w*/*w*).

**Figure 2:**
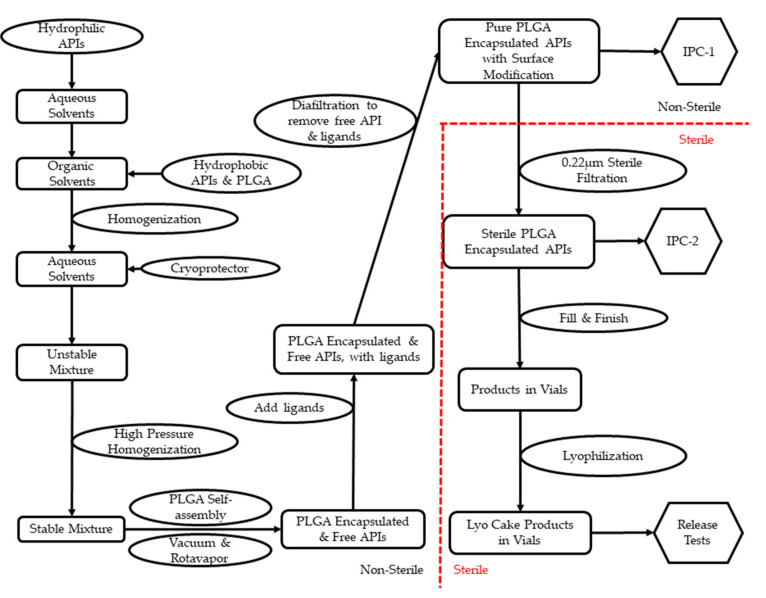
Concept flowchart of cGMP operations for PLGA NP-based drug products.

Figure 2 is a concept flowchart for cGMP production of PLGA NP-based pharmaceutical products. The steps in Figure 2 are provided on the basis of the details in Table 2 and the industry standards under cGMP guidelines.

## 6. Quality and Regulatory Assurance

The quality of nano-based pharmaceutical products is a learning curve for both manufacturers and regulatory agencies. In 2006, the FDA initialed the Nanotechnology Task Force to improve the service for nano-based products. After its first public report in 2007, FDA published the second report in 2020, emphasizing its commitment to nano-based products. Among the five published guidance for industry regarding nanomaterials, two of them were designed for pharmaceutical and medical products. Another draft guidance is under review for drug products and biological products. On the basis of the above documents, FDA will not accept or reject nanotechnology products according to category. Instead, FDA will conduct a comprehensive review to make a science-focused decision.

Size distribution is the FDA’s first consideration among all factors. Dynamic light scattering (DLS) technology is widely used for quality control purposes. Furthermore, a chromatography-based assay of APIs and encapsulation rate is critical to avoid toxicity. United States Pharmacopeia (USP) monograph “Goserelin Implants” also requests HPLC results to provide the retention time of PLGA, which is not rare for quality control of polymers [128]. Table 3 is a summary of the most needed in-process tests and final release tests.

During the Covid-19 pandemic, several liposome-based vaccines were approved for emergency use [129]. Liposome-based pharmaceutical products have similar self-assembly characteristics compared to PLGA NP-based ones. Therefore, their quality and regulatory focus should be similar. Furthermore, the success of vaccines also revealed the possibility of PLGA NP-based products to be approved through facilitated regulatory pathways, especially for emergency purposes under global regulatory systems [130,131].

## 7. Conclusions

This review presented recent progress in PLGA NPs as a vehicle to deliver drug to the brain in a controllable and targeted manner. Unlike most NPs, PLGA NPs show a promising future to become a clinically and commercially feasible drug delivery system. PLGA has been approved for both pharmaceutical products and medical devices, which provides clear quality control, quality assurance, and regulatory requirements for future products. Furthermore, the improvement of cGMP technologies, especially those in nanomedicines and sterile injectables, has also removed most obstacles for PLGA NPs. Even though safety is not the major concern, clinical trials are needed to monitor the efficacy and toxicity of PLGA NPs. Uncertainties, such as drug encapsulation rate, assembly stability, particle size distribution stability, and in vivo pharmacokinetics, may be the focus for future research and development.

## Figures and Tables

**Table 1:** Summary of BBB permeability studies of unmodified and modified PLGA NPs.

Ref.	PLGA NPs Modification	Loaded Drug	Proposed Transport Mechanism ^1^	Results
[67]	Trimethylated chitosan (TMC)	Coenzyme Q10 6-coumarin	AMT	TMC-modified PLGA NPs loaded with coumarin exhibited increased uptake in mouse brains vs. PLGA-NPs. Neuroprotective effects of Q10 displayed by mice in TMC PLGA NPs were superior to PLGA-NP.
[68]	Angiopep-2	Doxorubicin (DOX), Epidermal growth factor receptor (EGFR) siRNA	RMT	Angiopep-2 modified PLGA NPs improved DOX and siRNA cell uptake. In vivo study showed that the ang-2-PLGA construct can cross BBB.
[69]	8D3 monoclonal antibody	Loperamide	RMT	8D3 functionalized Loperamide-loaded PLGA NPs produced a higher maximal possible antinociceptive effect compared to Loperamide-loaded NPs without 8D3.
[70]	Lactoferrin, folic acid	Etoposide	RMT	BBB permeability coefficient of PLGA NPs increased twofold with Lf-and FA.
[71]	OX26 monoclonal antibody	1Aβ_5_ peptide	RMT	OX26 increased the uptake of PLGA NPs by BBB endothelial cells which enhanced the peptide transport.
[72]	Lactoferrin	Rotigotine	RMT	Intranasal delivery of rotigotine to the brain was more effective with Lf-PLGA NPs than with PLGA-NPs.
[73]	RVG29	Docetaxel	RMT	RVG29 PLGA NPs showed better BBB penetration in vitro.
[74]	OX26 monoclonal antibody	Temozolomide	RMT	OX26 functionalization enhanced TMZ internalization in glioblastoma cells.
[75]	Dendrimer cationized albumin	Doxorubicin	AMT	Cellular uptake and cell permeability of DOX in dCatAlb-functionalized PLGA NP was improved 1.59-fold and 1.49-fold, respectively, over unmodified PLGA NP.
[76]	D-α-tocopheryl polyethylene glycol succinate (TPGS)	Paclitaxel		The in vivo evaluation of TPGS–PLGA NPs showed amplified accumulation (>800% after 96 h) of PTX in the brain tissue when compared with bare NPs.
[77]	Polysorbate 80	Rhynchophylline	AMT	In an in vitro BBB model study, functionalized PLGA NPs showed increased transport across bEnd.3 cell monolayers.

^1^ Adsorption-mediated transcytosis (AMT), carrier-mediated transport (CMT), and receptor-mediated transcytosis (RMT).

**Table 2:** Processing details for PLGA NP-based systems from publications.

Ref	API	API Solvent	PLGA Solvent	Primary Aqueous	Secondary Aqueous	Purification
[117]	Doxorubicin	0.001 N HCl	Dichloromethane		1% PVA in PBS	
[118]	Transferrin	RH buffer (pH = 7.4)	Acetone/ethanol	Water		
[66]	Memantine/rhodamine	Water	Ethyl acetate	PVA solution	0.3% PVA	Centrifuge: 15,000 rpm, 20 min
[119]	Rhodamine-6G	PBS	Ethyl acetate		1% PVA	Centrifuge
[80]	Donepezil	Water	Dichloromethane	2% Pluronic F68	0.5% Pluronic F68	Centrifuge: 20,000× *g*, 20 min
[120]	Doxorubicin	Water	Dichloromethane	Water	1% PVA or 1% HSA PBS with pH 7.2	G2 sintered glass filter
[121]	Elaprase^®^	Water	Dichloromethane	Water	1% PVA	Centrifuge: 17,000 rpm, 10 min, 5 °C
[122]	Olanzapine	Acetonitrile	Acetonitrile	0.25% Poloxamer 407		Centrifuge: 25,000 rpm, 4 °C
[71]	iAβ5	Chloroform	Chloroform		0.1% Pluronic F127	Centrifuge: 14,300× *g*, 40 min
[123]	Curcumin	Ethyl acetate	Ethyl acetate	5% PVA	0.3% PVA	Centrifuge: 6000 rpm, 20 min
[124]	Thiazolidinedione	AcOEt/EtOH (80/20)	AcOEt/EtOH (80/20)	PBS with polysorbate 80		
[125]	Bacoside-A	Methanol/dichloromethane (1:2)	Methanol/dichloromethane (1:2)	2% PVA		Centrifuge: 13,000 rpm, 30 min, 4 °C
[126]	Curcumin	Acetonitrile	Acetonitrile	Lipids, EtOH in water		Centrifuge: 10 kDa
[89]	Curcumin, paclitaxel	Chloroform	Chloroform	1% sodium cholate	0.5% sodium cholate	
[127]	3,3′-Diindolylmethane	Ethyl acetate	Ethyl acetate	Didodecyldimethylammonium bromide		Centrifuge: 35,000 rpm, 1 h
[69]	Loperamide	Ethanol/ethyl acetate 20/80	Ethanol/ethyl acetate 20/80	PBS with polysorbate 80		Centrifuge: 3 kDa filter
[82]	Loperamide	Acetone/ethanol	Acetone/ethanol	0.1 wt.% TPGS	1 wt.% polysorbate 80 or 1 wt.% poloxamer 188	Centrifuge: 15,000× *g*, 1 h, 5 °C
[67]	Coenzyme Q10	Acetone	Acetone	0.02% vitamin E TPGS		Centrifuge: 15,000× *g*, 15 min, 20 °C

**Table 3:** In-process and release tests for PLGA NP-based drug products.

Purpose	USP Chapter	Test
IPC-1	N/A	High-performance liquid chromatography
	N/A	Particle size distribution
	N/A	Zeta potential
	<791>	pH
	N/A	Morphology
IPC-2	N/A	Filter integrity test
Release	<71>	Sterility
	<785>	Osmolality
	<467>	Residual organic solvents
	<281>	Residue on ignition
	<731, 921>	Loss on drying for lyophilized products
	<790>	Visible particulate inspection
	<61>	Microbial enumeration
	<791>	pH
	<85>	Bacterial endotoxins
	<788>	Particulate matter for injection
	<1207>	Uniformity of dosages

## Data Availability

Not applicable.
